# Supplementary material for: The relationship between ambivalence over the expression of emotions and somatic symptoms among Iranian long-distance and geographically close partners: The mediating role of emotional suppression
Source: PLOS Ment Health. 2025 Oct 15;2(10):e0000434. doi: 10.1371/journal.pmen.0000434 (PMC12798524; doi:10.1371/journal.pmen.0000434)
Supplement: S1 Text — Describing all variables. (DOCX) [file pmen.0000434.s003.docx]

This dataset contains anonymized responses from participants in the study of “The relationship between ambivalence over the expression of emotions and somatic symptoms among Iranian long-distance and geographically close partners: the mediating role of emotional suppression”. No identifying information is included. Below is a description of the variables:

**Demographic Variables**

- Age = Age in years
- Gender = Gender (1 = Female, 2 = Male)
- Type of Relationship = Long-distance relationship (LDR = 1) vs. Geographically close relationship (GCR = 2)
- Duration of relationship = Duration of relationship (1=Under 1 year, 2= 1-3 years, 3= 4-6 years, 4= 7-10 years, 5= more than 10 years).
- Education = Education level (1= Under diploma, 2= diploma, 3= bachelor, 4= master, 5= PhD and more)
- Children: Number of Children

**Questionnaire Variables**

- A1-A27= Items of the Ambivalence over Emotional Expression Questionnaire (AEQ) (Likert scale, 1–5)
- E1-E4= Items of the Emotion Regulation Questionnaire (ERQ) (Likert scale, 1–7)
- S1-S15= Items of the Patient Health Questionnaire (Likert scale, 1–3)
- L1- L12= Items of the Long-Distance Relationship Index (Likert scale, 1–7)

**Computed Scores**

- Positive Ambivalence= Total score of positive ambivalence
- Negative Ambivalence= Total score of negative ambivalence
- Tatal Ambivalence= Total score of ambivalence
- Emotional Suppression= Total score of Suppression subscale of ERQ
- Somatic Symptoms: Total somatic symptoms score
- Long Distance: Total scores of the Long-Distance Relationship Index

*Please note that all unanswered items in the dataset are coded as 999.
