# Supplementary material for: The relationship between ambivalence over the expression of emotions and somatic symptoms among Iranian long-distance and geographically close partners: The mediating role of emotional suppression
Source: PLOS Ment Health. 2025 Oct 15;2(10):e0000434. doi: 10.1371/journal.pmen.0000434 (PMC12798524; doi:10.1371/journal.pmen.0000434)
Supplement: Attachment — Submitted filename: Response to Reviewers.docx [file pmen.0000434.s005.docx]

**Point-by-point response to reviewer and editorial comments**

We thank the reviewers and the editor for their constructive feedback. We have revised the manuscript accordingly. Below we provide a point-by-point response (reviewer and editorial comments are in *italics and red*, our responses are in regular font(.

*Editorial Requests*

*Please provide separate figure files in .tif or .eps format.*

We have prepared and uploaded all figures as TIFF files in accordance with the journal’s requirements.

*Please ensure that your Ethics Statement is available in its entirety at the beginning of your Methods section, under a subheading 'Ethics Statement'.*

We thank the Editor for this reminder. We have now inserted a dedicated “Ethics Statement” subheading at the beginning of the Methods section, providing full details of the ethical approval (Approval ID: IR.SBU.REC.1402.078, Shahid Beheshti University)

*Please make all underlying data freely available in accordance with PLOS policies*

We thank the Editor for this important reminder. In compliance with PLOS data sharing policies, we have now provided full access to the anonymized dataset. The data have been uploaded as supplementary information (S1 Dataset) in both SPSS (.sav) and CSV formats, accompanied by a README file describing all variables. We have also updated the Data Availability Statement in the manuscript to reflect this change.

*Reviewer #1:*

*Lines 100- 101: Please provide the unit for the ages*

We thank the reviewer for this suggestion. We have clarified the unit for age as “years” in the text and in Table 1.

*Line 106: Was there a reason why you limited your participants to 45 years? Please shed light on this.*

We appreciate the reviewer’s insightful question. Participants were limited to the 18–45 age range for several reasons. First, excluding older adults reduced the potential confounding effect of age-related increases in somatic symptoms, ensuring that observed associations were not attributable to general health concerns associated with aging. Second, access to social media and online platforms is more prevalent in this group, which was essential for recruitment and for completing the online questionnaire. Finally, given the recent migration trends in Iran, individuals in this age group are more likely to be engaged in long-distance relationships, making them the most relevant population for this study.

*Lines 116-119: Was there any benchmark you used in categorizing the participants to LDRs and GCRs? If there were, please state them for the readers.*

We thank the reviewer for raising this question. We clarify that the Long-Distance Romantic Relationship Index does not provide a standardized cut-off point for categorizing participants. Therefore, consistent with the data collected in our study, we used the sample mean as the threshold to classify participants into LDR and GCR groups. We have now made this explanation explicit in the Participants section and acknowledged the potential limitation of this approach in the Limitations section.

*Line 146: ‘Questionnaire’ should be ‘questionnaire’*

We thank the reviewer for pointing this out. We have revised the capitalization and changed “Questionnaire” to “questionnaire”.

*Line 158: The numbering of your themes and sub-themes, such as ‘2.3.4’ in the manuscript, is not necessary. It seems that you are formatting a thesis for publication. Please delete the numbers for consistency in this journal.*

We thank the reviewer for pointing this out. We have removed the numbering of themes and sub-themes (e.g., “2.3.4”) and reformatted them according to the journal style.

*Line 195: Please revise your table title to: Participants’ demographic and descriptive statistics*

We thank the reviewer for this helpful comment. We have revised the title of Table 1 to “Participants’ demographic and descriptive statistics” as suggested.

*Line 195: In the table, revise the unit of age as ‘years’*

We thank the reviewer for this comment. We have revised the table to indicate the unit of age as “years” in Table 1.

*Line 197: Please include the age unit*

We thank the reviewer for this helpful comment. We have revised the manuscript to consistently include the unit of age as “years” in both the text and the tables.

*Line 198: Please change ‘Chi2’ to ‘X2’, or get the actual symbol*

We thank the reviewer for this comment. We have revised the manuscript and replaced “Chi2” with the correct symbol “χ²” throughout the text and tables.

*Line 201: Please insert the time unit*

We thank the reviewer for pointing this out. The “time spent together” variable was not measured in hours or days, but rather using a Likert-type item (1 = very little, 7 = very much) from the Long-Distance Romantic Relationship Index. We have revised the Results section to clarify that the reported values represent mean scores on this scale, not actual time units.

*Lines 347- 350: ‘This challenges common assumptions….’, you would need to scale down the tone of your argument because of your study design (cross-sectional) and sampling technique (convenience).*

We thank the reviewer for this important point. We have revised the sentence to scale down the tone of our claim. Instead of stating that our findings “challenge common assumptions,” we now describe them as “preliminary evidence” that may question such assumptions, and we explicitly note that these conclusions must be interpreted with caution given our cross-sectional design and convenience sampling.

*Lines 338- 369: Your conclusion is too long. Kindly abridge it.*

We thank the reviewer for this constructive suggestion. We have abridged the Conclusion section to make it more concise and focused on the main findings, their implications, and future directions, while avoiding repetition of details already presented in the Discussion.

*Line 370: Also, include your study design (cross-sectional) as a limitation.*

We thank the reviewer for this comment. We have added a statement in the Limitations section to acknowledge that the study’s cross-sectional design prevents causal interpretations and longitudinal inferences.

*Reviewer #2: Introduction*

*You mentioned "few studies have explored these relationships in LDRs versus GCRs, particularly in non-Western populations" (lines 83), but you could clarify why Iranian couples are especially important to study (e.g., cultural norms around emotion expression, relationship expectations, or stressors etc.) It will provide a robust reason behind conducting the study.*

We thank the reviewer for this helpful comment. We have now expanded the Introduction to clarify why studying Iranian couples is important. Previous LDR research has predominantly relied on homogeneous samples from Western, individualistic societies, mostly White middle-class couples. This narrow scope risks overlooking cultural norms that influence attachment and relational expectations. By examining LDRs in a collectivist context such as Iran, our study contributes culturally grounded insights and highlights the possibility that relationship dynamics may differ from those observed in Western populations.

*Participants and Procedure*

*The phrase “The data were accessed from June 1, 2023” (line 110) is unclear. Likely meant “data collection began June 1, 2023?” because in line 105 you have mentioned that you began to collect data. Please rephrase it or clarify or remove the sentence.*

We thank the reviewer for pointing this out. The unclear sentence has been removed, and we now clearly state: “Data were collected using an online questionnaire from June 2023 to January 2024.”

*Please mention how many missing data were there before cleaning.*

We thank the reviewer for this point. Since all items in our online questionnaire were set as mandatory, no missing data were observed. We have now clarified this in the Participants and Procedure section of the manuscript.

*Acknowledgement*

*please acknowledge the coauthors and their contribution. Also acknowledge the research assistants who worked for the study but could not be added as coauthors in the study*

We appreciate this comment. We have now revised an Acknowledgements section to the manuscript. In it, we thank all co-authors for their contributions to the design, analysis, and writing of the study. As the study did not involve additional research assistants, we also acknowledged the participants whose cooperation made the project possible.

We thank the Editor and Reviewers again for their constructive feedback and helpful suggestions, which have significantly improved our manuscript.
